# Pathogenicity and molecular evolutionary analysis of novel *Avian orthreovirus* genotypes IV and V in Upper Egypt

**DOI:** 10.1016/j.virusres.2026.199779

**Published:** 2026-07-20

**Authors:** Doha Abd Alrahman Ahmed, Shimaa Mohamed Ali Ahmed Khair, Mohammed A. GamalEldin, Ibrahim Mohamed Eldaghayes, Eman Abd Elmenum Shosha

**Affiliations:** a Department of Avian and Rabbit Medicine, Faculty of Veterinary Medicine, Assiut University, Assiut, Egypt; b Department of Poultry and Rabbit Medicine, Faculty of Veterinary Medicine, Sphinx University, Assiut, Egypt; c Department of Poultry Diseases, Assiut Provincial Laboratory, Animal Health Research Institute, Agricultural Research Center (ARC), Assiut, Egypt; d Department of Microbiology and Parasitology, Faculty of Veterinary Medicine, University of Tripoli, P.O. Box 13662, Tripoli, Libya; e Virology Department, Faculty of Veterinary Medicine, New Valley University, El-Khargia, Egypt

**Keywords:** *Avian orthoreovirus*, Genotype IV, Genotype V, Upper Egypt, σC gene, Pathogenicity, Vaccine mismatch

## Abstract

*Avian orthoreovirus* (ARV) imposes substantial economic losses on the global poultry industry, primarily through runting–stunting syndrome and viral arthritis. Despite its global prevalence, the molecular epidemiology and pathogenic landscape of ARV strains circulating in Upper Egypt remain largely unexplored. This study investigated the molecular characteristics, phylogenetic relationships, and pathogenic potential of ARV in broiler flocks across Assiut, Sohag, and New Valley governorates. Out of 140 clinical samples from chickens showing stunted growth and tendon swelling, 42 (30%) were positive by RT-qPCR targeting the σC gene. Phylogenetic analysis of 11 representative isolates using MEGA X revealed a complex co-circulation of two genetically distinct genotypes. Notably, this study provides the first report of ARV Genotypes IV (GC IV) and V (GC V) in Upper Egypt governorates. Nine isolates clustered within Genotype V, forming a unique regional sublineage, while two isolates belonged to Genotype IV, showing high homology to contemporary Asian strains. Crucially, these field isolates exhibited extreme genetic divergence from commercial vaccine strains, sharing as little as 38% amino acid identity, indicating a severe vaccine mismatch. Experimental infection of young chicks confirmed high virulence, characterized by significantly impaired growth, reduced tibial length, and elevated feed conversion ratios (p <0.05). High viral loads were quantified in the tendons, pancreas, and proventriculus (p < 0.01). These findings underscore the urgent emergence of novel ARV variants in Upper Egypt and highlight the critical need for developing regionally-tailored vaccination strategies to mitigate these evolving threats.

## Highlights

• This study provides the first comprehensive molecular and pathogenic characterization of Avian Reovirus (ARV) Genotypes IV and V in Upper Egypt for the first time as it is the first record, using various methods.
• Notably, this research represents the exclusive isolation and characterization of ARV from broiler flocks in this specific geographical region (Assiut, Sohag, and New Valley), where no previous virological studies of this nature have been conducted.
• Our findings reveal a dramatic genetic divergence (up to 62% in amino acid identity) between the circulating field strains and the currently implemented vaccine strains (S1133).
• These results highlight a critical vaccine mismatch and underscore an urgent need for regionally adapted vaccination strategies to mitigate the severe economic impact on the poultry industry.

## 1 Introduction

*Avian orthoreovirus (ARV)* remains a critical global concern in poultry production, associated with considerable economic losses and reduced performance in commercial flocks worldwide. *Avian orthoreovirus* infections are among the most significant viral diseases affecting poultry, causing arthritis, tenosynovitis, malabsorption syndrome, and growth retardation, thereby causing detrimental financial losses in the poultry industry worldwide (Al-Ebshahy et al., 2020; Jiang et al., 2023). Taxonomically, according to the standard International Committee on Taxonomy of Viruses (ICTV) framework, avian *orthoreovirus* belongs to the family *Sedoreoviridae (*formerly *Reoviridae*), subfamily *Spinareovirinae*, and genus *Orthoreovirus* (Zhang et al., 2019). Consequently, sequencing of the **σC** gene has become the primary molecular tool for ARV genotyping and epidemiological investigations. Based on phylogenetic analyses of σC gene, circulating Avian orthoreovirus isolates worldwide are currently classified into at least six major genotypes I–VI (six genotypic clusters GCI–GCVI). Classical reference strains, including the extensively used vaccine strain S1133, are consistently belonging to genotype I, while the remaining genotypes comprise genetically diverse field strains identified across different geographic regions (Rafique et al., 2024**;** Kariithi et al., 2026).

The virus is non-enveloped with 10 double-stranded RNA genome segments that encode four nonstructural proteins (p10, p17, μNS, σNS) and eight structural proteins (μA, μB, λA, λB, λC, σA, σB, σC) (Wang et al., 2023). Among these, the highly variable *S1* gene segment, encoding the σC protein, is pivotal for viral attachment, the induction of neutralizing antibodies, and serotype differentiation (Nour and Mohanty, 2024). Consequently, the *S1* gene is regarded as the gold standard for the molecular characterization and phylogenetic assessment of circulating ARV strains (Chen et al., 2020).

ARV infections manifest through diverse clinical presentations, including tenosynovitis/viral arthritis (VA), runting-stunting syndrome (RSS), malabsorption syndrome (MAS), and secondary immunosuppression (Choi et al., 2022). Pathologically, the virus targets the joints, tendons, and intestinal tract, resulting in lameness, hock joint swelling, and marked growth retardation (Jiang et al., 2023, Mosad et al., 2023). Transmission occurs via both horizontal (fecal–oral) and vertical routes (from infected breeders to progeny) (Jiang et al., 2023, Rafique et al., 2024). Despite the intensive application of attenuated or inactivated vaccines, field protection is frequently compromised by extensive antigenic diversity and the continuous emergence of novel variants (Lu et al., 2015, Yu et al., 2025).

In Egypt, ARV infections have evolved into a major biosecurity threat, characterized by recurrent outbreaks even in strictly vaccinated flocks (Al-Ebshahy et al., 2020). Recent molecular surveillance has identified a complex epidemiological landscape involving the co-circulation of multiple lineages, predominantly Genotypes II, IV, and V (Zanaty et al., 2023, Arafa et al., 2024). Notably, the emergence of Genotype V variants in Egyptian poultry has been linked to severe "vaccine breakthroughs." These field strains exhibit significant antigenic drift and limited genetic homology with traditional vaccinal strains (Zanaty et al., 2023). Such genetic divergence underscores the insufficient cross-protection provided by current vaccines, necessitating a transition toward autogenous or regionally tailored vaccination strategies (Arafa et al., 2024, Mohamed et al., 2020). While many studies in Egypt have focused on the northern provinces, a substantial knowledge gap remains regarding the molecular evolution of ARV in Upper Egypt governorates. Despite the rapid expansion of the poultry sector in regions like Assiut and Sohag, comprehensive molecular and pathogenetic surveillance is still lacking. Furthermore, recent outbreaks in these areas suggest an escalating divergence of field strains. Therefore, the present study aimed to molecularly and pathogenetically characterize ARV strains isolated from broiler chickens in Upper Egypt governorates during 2024–2025 and to evaluate the phylogenetic relationships between these isolates and commercial vaccine strains based on the *S1* gene.

## 2 Materials and methods

### 2.1 Ethical approval

The protocols for the use and care of laboratory animals were established by the Faculty of Veterinary Medicine at Assiut University in Egypt. Approval Number: [06/2025/0374] on 20 October 2025. The Institutional Animal Care and Use Committee of Assiut University examined and approved the experimental protocol. To minimize animal distress, all birds were humanely anesthetized using an appropriate dose of a ketamine/xylazine combination (25/5 mg/kg BW) before any invasive handling or sampling. At the conclusion of the study, using cervical dislocation, birds were humanely put to death following deep general anesthesia, ensuring strict adherence to international animal welfare standards.

### 2.2 Sampling

Between 2023 and 2024, a total of 140 tissue samples (including synovial fluid, gastrocnemius tendons, and proventriculus) were collected from 50 independent commercial broiler flocks located in Upper Egypt (Assiut, Sohag, and New Valley Provinces). These provinces were selected to provide a comprehensive representation of diverse poultry production systems in the region. From each flock, birds were selected based on clinical signs of varying severity, including lameness (scoring 2–3 on a modified gait scale) and stunting (body weight reduction of >20% compared to the flock average). The sampled broilers originated from breeder flocks previously immunized with a standard S1133-based vaccination program (Cluster I, GCI), consisting of one live attenuated dose followed by three inactivated doses. To investigate tissue-specific viral distribution, synovial fluid and gastrocnemius tendon tissues were harvested from birds exhibiting lameness, while proventriculus samples were collected from birds showing poor growth performance. From the 140 samples collected (50 synovial fluid/tendons and 90 proventriculus), every positive isolate used for subsequent molecular characterization represented a distinct flock to ensure epidemiological independence. All samples were stored at -80°C in 50% buffered glycerin until processing (York and Fahey, 1988).

### 2.3 Specimen processing and virus isolation

Frozen tissue samples were thawed and homogenized in sterile phosphate buffer saline (PBS) to prepare a 10%–20% (w/v) suspension. Homogenates were clarified by centrifugation at 3,000 rpm for 30 mins. Supernatants were treated with an antibiotic-antifungal cocktail (2,000 units/mL penicillin, 2 mg/mL streptomycin, 50 µg/mL gentamicin, and 1,000 units/mL mycostatin) for 1 h at room temperature. For virus isolation, 0.2 mL of the clarified supernatant was inoculated into the yolk sac of 7-day-old specific pathogen-free embryonated chicken eggs (SPF-ECEs), according to standard procedures (Mansour et al., 2018). Inoculated eggs were incubated at 37°C and monitored daily for 7 days. To ensure successful virus adaptation and propagation, three successive passages were performed in SPF-ECEs. For each passage, the yolk sac membranes and fluids were harvested from inoculated eggs after undergoing two consecutive freeze-thaw cycles at -80°C to ensure maximum viral release, followed by clarification via centrifugation. ARV-positive samples were identified by embryo mortality or characteristic lesions, such as hemorrhages and liver congestion, occurring 3–5 days post-inoculation (Mansour et al., 2018**,** Lu et al., 2015). Specificity was confirmed by testing the harvested yolk sac membranes via real-time PCR (RT-qPCR).

### 2.4 Molecular identification

Total RNA was extracted from the materials of the third egg passage (specifically the clarified yolk sac fluids and harvested yolk membranes representing the isolated virus) using the QIAamp MinElute Virus Spin Kit (Qiagen, Hilden, Germany), according to the manufacturer’s protocol. For initial screening and differentiation between field and vaccine strains, the Kylt® Avian orthoreovirus RT-qPCR kit and the Kylt® Avian orthoreovirus S1133 DIVA kit (AniCon, Germany) were employed; both kits target the σC C gene to ensure accurate genotyping. The RT-qPCR was performed in a 20 µL reaction volume containing 5 µL of template RNA, using a QuantStudio 5 Real-Time PCR System (Applied Biosystems, USA). Thermal cycling conditions followed the PCR kit manufacturer's instructions, and samples with a cycle threshold (Ct) value < 30 (as per the kit’s diagnostic cutoff) were considered positive for ARV. For molecular characterization, samples were initially screened using conventional RT-qPCR targeting the σC gene; this target was specifically selected as a DIVA (Differentiating Infected from Vaccinated Animals) strategy to distinguish field isolates from vaccine strains from the outset. Subsequently, for detailed molecular characterization, conventional RT-PCR targeting the same σC-encoding region of the S1 gene was conducted on selected samples with high viral loads (Ct < 30). The reaction was performed using the AgPath-ID™ One-Step RT-PCR kit (Applied Biosystems, USA) in a 25 µL reaction mixture consisting of 12.5 µL of 2X RT-PCR buffer, 1 µL of 25X RT-PCR enzyme mix, 0.4 µM of each primer (P1: 5′-AGTATTTGTGAGTACGATTG-3′ and P4: 5′-GGCGCCACACCTTAGGT-3′), and 5 µL of template RNA. These primers were utilized to amplify a specific 790 bp internal fragment of the σC gene, focusing on the hypervariable region to ensure high-quality sequencing and accurate genotyping. Thermal cycling was conducted in an [Applied Biosystems™ 7500] following the kit manufacturer’s optimized protocol (Kant et al., 2003). Amplification was carried out in a Biometra thermal cycler (T Gradient, Germany) with the following parameters: reverse transcription at 50°C for 30 min; initial denaturation at 94°C for 2 min; 35 cycles of 94°C for 1 min, 58°C for 1 min. (annealing), and 72°C for 1 min; and a final extension at 72°C for 10 min. The resulting 790 bp amplicons were resolved by electrophoresis on a 1.5% (w/v) agarose gel stained with ethidium bromide and visualized under UV light. ARV S1133 (OQ675704, MEVAC) and RNase-free water served as positive and negative controls, respectively.

### 2.5 Sequencing, phylogenetic analysis, and recombination events

For further molecular characterization, the PCR products exhibiting the highest band intensity were purified using the QIAquick Gel Extraction Kit (QIAGEN, Germantown, MD, USA). Eleven representative purified DNA samples were submitted to Macrogen (Seoul, South Korea) for bidirectional sequencing using the P1 and P4 primer set. The raw sequence data were trimmed and assembled using SeqMan software (DNASTAR, Madison, WI, USA). The resulting sequences were verified using NCBI BLAST and deposited in GenBank under accession numbers: PX252141–PX252143 and PX609969–PX609976.

Multiple sequence alignments (MSA) for both nucleotide and amino acid sequences were performed using ClustalW within BioEdit version 7.1 (Hall, 1999). Phylogenetic trees were constructed using MEGA X software. To ensure the most accurate evolutionary relationship, the Neighbor-Joining (NJ) method was applied, and evolutionary distances were computed using the Kimura 2-parameter model. The robustness of the phylogenetic branches was evaluated by bootstrap analysis with 1000 replicates. The reference strains used for comparison included global and local ARV genotypes (Genotypes I–VI) to determine the lineage of the Upper Egypt isolates.

Recombination events were identified using RDP v4.5 software. The detected events were further assessed by analytical tool of BootScan, and 3Seq. A p-value threshold of ≤0.05 was applied to confirm the significance of the recombination areas. In addition, the relationships between the putative parental sequences involved in each recombination event were investigated following the identification of recombination regions (Wilson and McVean, 2006).

### 2.6 Pathogenicity, performance impact of the isolated ARV Strain, and co-infection

A total of sixty 1-day-old specific pathogen free (SPF) chicks, free of avian pathogens, were purchased from Kom-Osheim farm, Fayoum governorate of Egypt (Ma et al., 2025). The chicks were randomly assigned to two groups (n= 30 for an infection group and 30 for a control group). This sample size was justified based on a statistical power analysis (α = 0.05, power = 0.80) to ensure sufficient sensitivity in detecting significant differences in growth performance (Wei et al., 2025). On day 1, the challenged group was inoculated via footpad injection with 0.1 mL of the local purified ARV isolate (ARV- New valley- vet-Doha-PX252143/2025 of genotype IV) at a titer of 10^6^ EID_50_/mL (determined by titration in SPF embryonated chicken eggs), while the control group received an equal volume of sterile PBS via the same route (Jiang et al., 2023**,** Chen et al., 2025).

The birds were housed in separate positive-pressure isolators under strict biosecurity and monitored daily for clinical manifestations, including tenosynovitis/arthritis, lameness, and runting-stunting syndrome. Growth performance was assessed by recording individual body weights at 1, 7, 14, 21, 28, 35, and 42 days of age. Feed intake was monitored to calculate the feed conversion ratio (FCR) at 42 days. Skeletal development was evaluated by measuring tibial length weekly from 14 to 42 days. To assess lesion progression and viral dynamics, three birds per group were randomly selected and humanely euthanized at 7, 14, 21, 28, and 35 days post-infection (dpi). At the conclusion of the experiment (day 42), all remaining birds were euthanized and examined for pathognomonic gross lesions. Viral load quantification was performed via one-step RT-qPCR targeting the σC gene in the harvested tendon tissue (Chen et al., 2025).

To ensure the isolates were free from co-infecting agents, the viral stocks underwent rigorous molecular and bacteriological screening. All isolates were confirmed negative for major avian viral pathogens, including *Avian Influenza Virus* (AIV; M-gene and H5 subtype), Newcastle Disease Virus (NDV), *Infectious Bursal Disease Virus* (IBDV), *Marek’s Disease Virus* (MDV), *Chicken Infectious Anemia Virus* (CAV), *Fowl Adenoviruses* (FAdVs), *Avian Encephalomyelitis Virus* (AEV), and *Chicken Astrovirus* (CAstV) using specific RT-qPCR/PCR assays. Furthermore, bacteriological purity was verified by the absence of common poultry pathogens, including Salmonella species (invA), *Escherichia coli* (phoA), *Clostridium perfringens* (Net-B), Mycoplasma species (MSv), *Staphylococcus aureus* (nuc), and Streptococcus species (pbp1A**)** (Table 1).

**Table 1:** Consensus primer and probes sets applied for the identification of other co-infected viruses in collected samples.

ID	Primer and probe sequences	Reference
AIV-M-gene	sep1:AGATGAGTCTTCTAACCGAGGTCG sep2:TGCAAAAACATCTTCAAGTCTCTG sep-probe: FAM-TCAGGCCCCCTCAAAGCCGA-TAMRA	(Mirzaiee et al., 2020)
AIV-H5 subtype	H5LH1:ACATATGACTACCCACARTATTG H5RH1: AGACCAGCTAYCATGATTGC H5PRO: FAM-TCWACAGTGGCGAGTTCCCTAGCA-TAMRA	(Abdelwhab el-SM et al., 2010; Shosha et al., 2025a)
IBV	AIBV-fr: ATGCTCAACCTTGTCCCTAGCA AIBV-as: TCAAACTGCGGATCATCACGT AIBV-TM: FAM-TTGGAAGTAGAGTGACGCCCAAACTTCA-TAMRA	(Shosha et al., 2025b)
NDV	F +4839: TCCGGAGGATACAAGGGTCT F -4939: AGCTGTTGCAACCCCAAG F +4894: FAM-AAGCGTTTCTGTCTCCTTCCTCCA-TAMRA	(Wise et al., 2004)
IBDV	F/AUS GU: TCACCGTCCTCAGCTTACCCACATC R/AUS GL: GGATTTGGGATCAGCTCGAAGTTGC	(Mosad et al., 2024)
MDV	MDV-F: GGCACGGTACAGGTGTAAAGAG MDV-R: GCATAGACGATGTGCTGCTGAG	(Cheng et al., 2024)
CAV	CAV-F: GGTACGTATAGTGTGAGGC CAV-R: GCTGTGAGTGTTGCAAAGCT	(Liu et al., 2025; Soliman et al., 2026; Shosha et al., 2026a)
FAdVs	F- 5ʹ-ACATGGGAGCGACCTACTTCGACA-3ʹ R- 5ʹ-TCGGCGAGCATGTACTGGTAAC-3ʹ.	(Adel et al., 2021; Shosha et al., 2026b)
AEV	-F:5’GAATTAGCTCCTGGTAAACCTCG-3’ R: 5’-CTCTATCGCAACACCCTCAGG-3’	(Zhang et al., 2024; Shosha et al., 2026c)
CAstV	Fast-deg-2 5′- GCA TGG CTC CAC CGT AAG C -3′ Rast-2 5′- ACA CTC CCA GCA ACA TTT G -3′	(Sallam et al., 2023)
*E. coli phoA*	F- 5ʹ-CGATTCTGGAAATGGCAAAAG-3ʹ R- 5ʹ-CGTGATCAGCGGTGACTATGAC	(Nahar et al., 2018; Ahmed et al., 2026)
*Salmonella invA*	F- 5ʹ-GTGAAATTATCGCCACGTTCGGGCAA-3ʹ R- 5ʹ-TCATCGCACCGTCAAAGGAACC-3ʹ	(Palmeira et al., 2016)
Clostridia peregrines Net-B toxin gene	F- 5ʹ-GCTGGTGCTGGAATAAATGC-3ʹ R- 5ʹ-TCGCCATTGAGTAGTTTCCC-3ʹ	(Datta et al., 2014; Ahmed et al., 2025)
Mycoplasma MSv gene	F: 5’-GGC CAT TGC TCC TRC TGT TAT-3’ R: 5’-AGT AAC CGA TCC GCT TAA TGC-3’	(Rufai et al., 2025)
*Staphylococcus aureus* (*nuc*) gene	F: 5’GCGATTGATGGTGATACGGT-3’ R:5’AGCCAAGCCTTGACGAACTAAAG3’	(Brakstad et al., 1992)
*Streptococcus* spp. *pbp*1A gene	F: 5’*AAACAAGGTCGGACTCAACC*-3’ R: 5’*AGGTGCTACAAATTGAGAGG*-3’	(Wódz et al., 2024)

### 2.7 Statistical analysis

Statistical analyses were performed using GraphPad Prism software version 10.0. The normality of data distribution was assessed using the Shapiro–Wilk test, and the homogeneity of variances was confirmed by Levene’s test. The statistical units were defined as follows: the individual bird for body weight and tibia length; the replicate (n=3 replicates per group, with birds subdivided within the isolator) for Feed Conversion Ratio (FCR); and the individual sampled bird for viral load quantification (n=3 per time point). For data involving multiple time points (body weight and tibia length), a two-way ANOVA was conducted using group and time as the main factors. Single-point comparisons, such as cumulative FCR, were analyzed using the unpaired Student’s t-test. Following ANOVA, Tukey’s Honestly Significant Difference (HSD) test was employed for post-hoc multiple comparisons. All data are presented as Mean ± Standard Error (SE). A P-value < 0.05 was considered statistically significant.

## 3 Results

### 3.1 Clinical findings and gross lesions in field cases

**Fig. 1:**
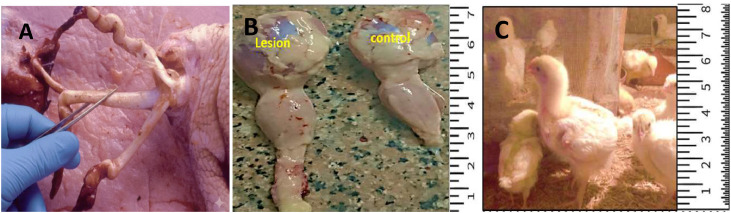
Field suspected broiler chickens showing clinical and gross lesions associated with Avian orthoreovirus (ARV) infection. (A) Bird showing severe tenosynovitis and rupture of the gastrocnemius tendon; (B) Markedly dilated proventriculus; (C) Stunted and unevenly grown birds within the same flock.

The broiler chickens examined during the study period exhibited characteristic clinical signs consistent with viral arthritis and runting-stunting syndrome (RSS). Affected birds showed varying degrees of lameness, reluctance to move, and hock swelling. In severe cases, rupture of the gastrocnemius tendon was observed in 40-day-old broilers, resulting in a typical “hockey stick” leg appearance (green hock). Birds with RSS exhibited stunted growth, uneven body weights, and poor feed conversion compared with unaffected flocks. Upon necropsy, the internal organs revealed pathognomonic lesions; the intestines appeared pale, distended, and contained undigested feed particles, indicating severe malabsorption. Notably, the pancreas showed marked atrophy and pale discoloration with occasional necrotic foci. Furthermore, the affected hock joints exhibited serosanguinous synovial fluid and thickening of the digital flexor tendons (Fig. 1).

### 3.2 Molecular ARV detection

**Fig. 2:**
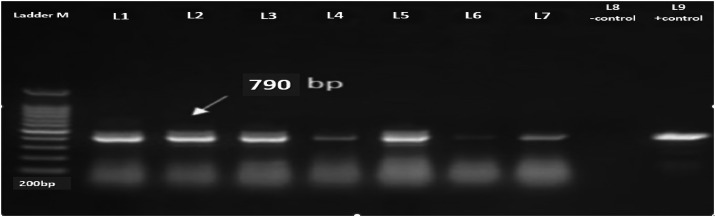
Agarose gel electrophoresis of RT-PCR products targeting the σC gene of Avian orthoreovirus (ARV). Lane M: 200 bp DNA ladder; lanes 1–7: positive field samples showing the expected 790 bp amplicon; lane 8: negative control (nuclease-free water); lane 9: positive control (ARV S1133 strain).

Of the 140 examined tissue samples, 42 (30%) tested positive for Avian orthoreovirus (ARV) RNA using the Kylt® AVR RT-qPCR assay. These positive samples originated from 35 out of 50 (70%) investigated flocks. The tissue-specific distribution of the viral RNA revealed that 30 out of 90 (33.3%) proventriculus samples were positive, while 12 out of 50 (24%) synovial fluid and gastrocnemius tendon samples tested positive. Samples with low Ct-values (<30) were selected for further confirmation by conventional RT-PCR targeting the σC gene-encoding region of S1 gene. The reaction produced a distinct amplicon of approximately 790 bp, confirming the presence of ARV genomic RNA (Fig. 2).

### 3.3 Sequencing and GenBank submission

Eleven representative RT-PCR–positive samples, four from Assiut, four from Sohag, and three from New Valley provinces, were successfully sequenced. The sequences were deposited in GenBank under the following accession numbers: PX252141–PX252143 and PX609969–PX609976 (Table 2). All accession numbers were verified for public accessibility and consistency across the manuscript.

**Table 2:** Data for eleven Egyptian Avian orthoreovirus (ARV) isolates analyzed in this study.

Num-ber	Isolates	Age/Day	Isolat-ion date	Clinical Signs	tissues isolated	Host	Vaccination status of breeder	Geoloc-ation /Province	genotype cluster	Accession number
1	Assiut-vet-Doha	14	2025	Growth retardation, poor feathering	Proventri-culus, pancreas-e	Broiler chickens	Yes	Assiut	Cluster V	PX252141
2	Assiut_vet_Doha1	20	2025	Arthritis	Tendon sheath, tendon	Broiler chickens	Yes	Assiut	Cluster V	PX609969
3	Assiut_vet_Doha2	25	2025	Growth retardation	Proventri-culus, pancreas-e	Broiler chickens	Yes	Assiut	Cluster V	PX609970
4	Assiut_vet_Doha3	12	2025	Arthritis, growth retardation, poor feathering	Proventri-culus, pancreas-e Tendon sheath	Broiler chickens	Yes	Assiut	Cluster V	PX609971
5	Sohag_vet_Doha	13	2025	Growth retardation, poor feathering	Proventri-culus, pancreas-e	Broiler chickens	Yes	Sohag	Cluster IV	PX252142
6	Sohag_vet_Doha1	27	2025	Arthritis	Tendon sheath, tendon	Broiler chickens	Yes	Sohag	Cluster V	PX609972
7	Sohag_vet_Doha2	11	2025	Arthritis, growth retardation, poor feathering	Tendon sheath, tendon	Broiler chickens	Yes	Sohag	Cluster V	PX609973
8	Sohag_vet_Doha3	35	2025	Arthritis	Proventri-culus, pancreas-e	Broiler chickens	Yes	Sohag	Cluster V	PX609974
9	New valley_vet_Doha	10	2025	Arthritis, growth retardation, poor feathering	Tendon sheath, tendon	Broiler chickens	Yes	New valley	Cluster IV	PX252143
10	Newvalley_vet_Doha1	20	2025	Growth retardation, poor	Tendon sheath, tendon	Broiler chickens	Yes	New valley	Cluster V	PX609975
11	Newvalley_vet_Doha2	30	2025	Arthritis	Proventri-culus, pancreas-e	Broiler chickens	Yes	New valley	Cluster V	PX609976

### 3.4 Phylogenetic analysis

**Fig. 3:**
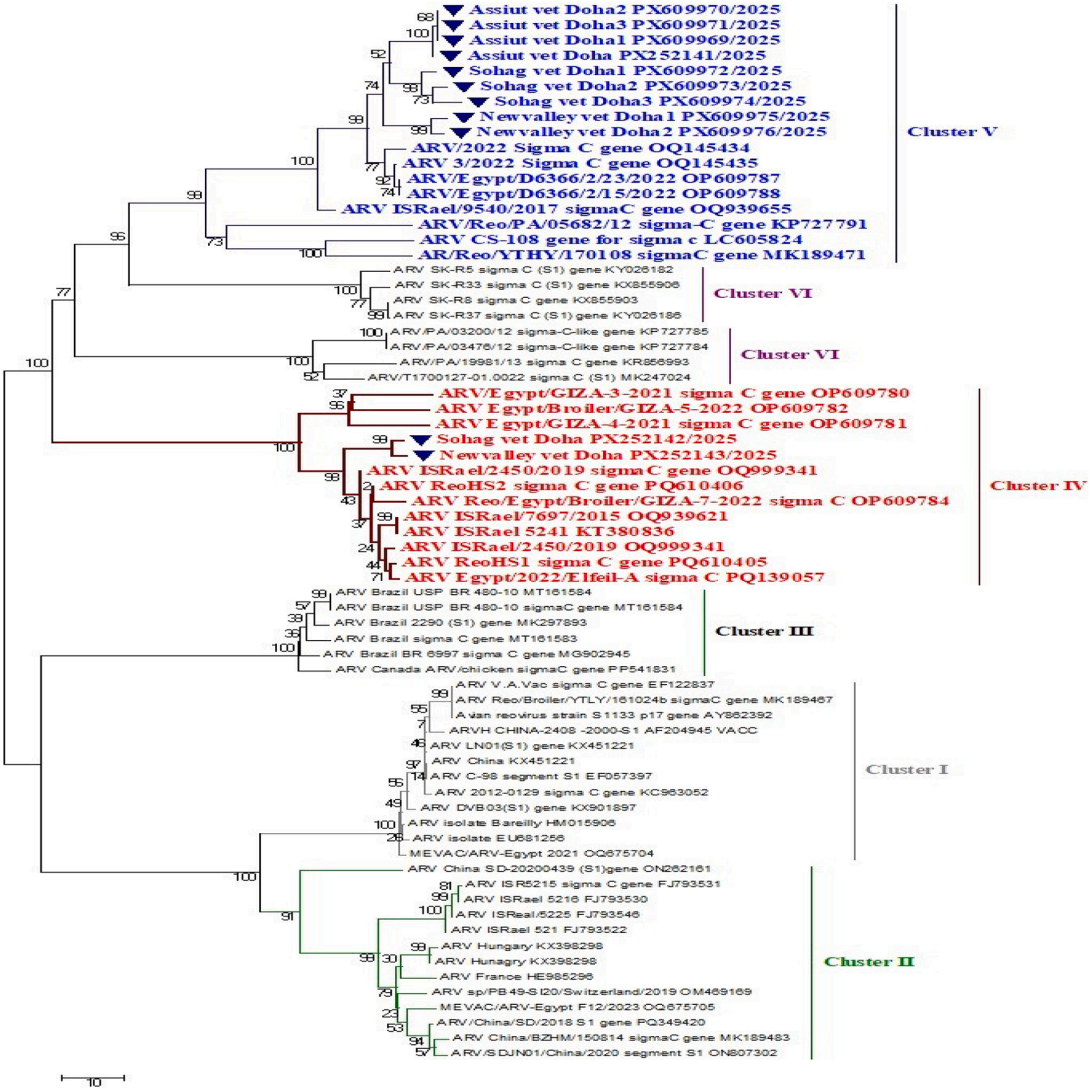
Phylogenetic tree based on partial nucleotide sequences of the σC gene of Avian orthoreovirus isolates. The tree was constructed using the neighbor-joining method (MEGA X, 1000 bootstrap replicates).

**Fig. 4:**
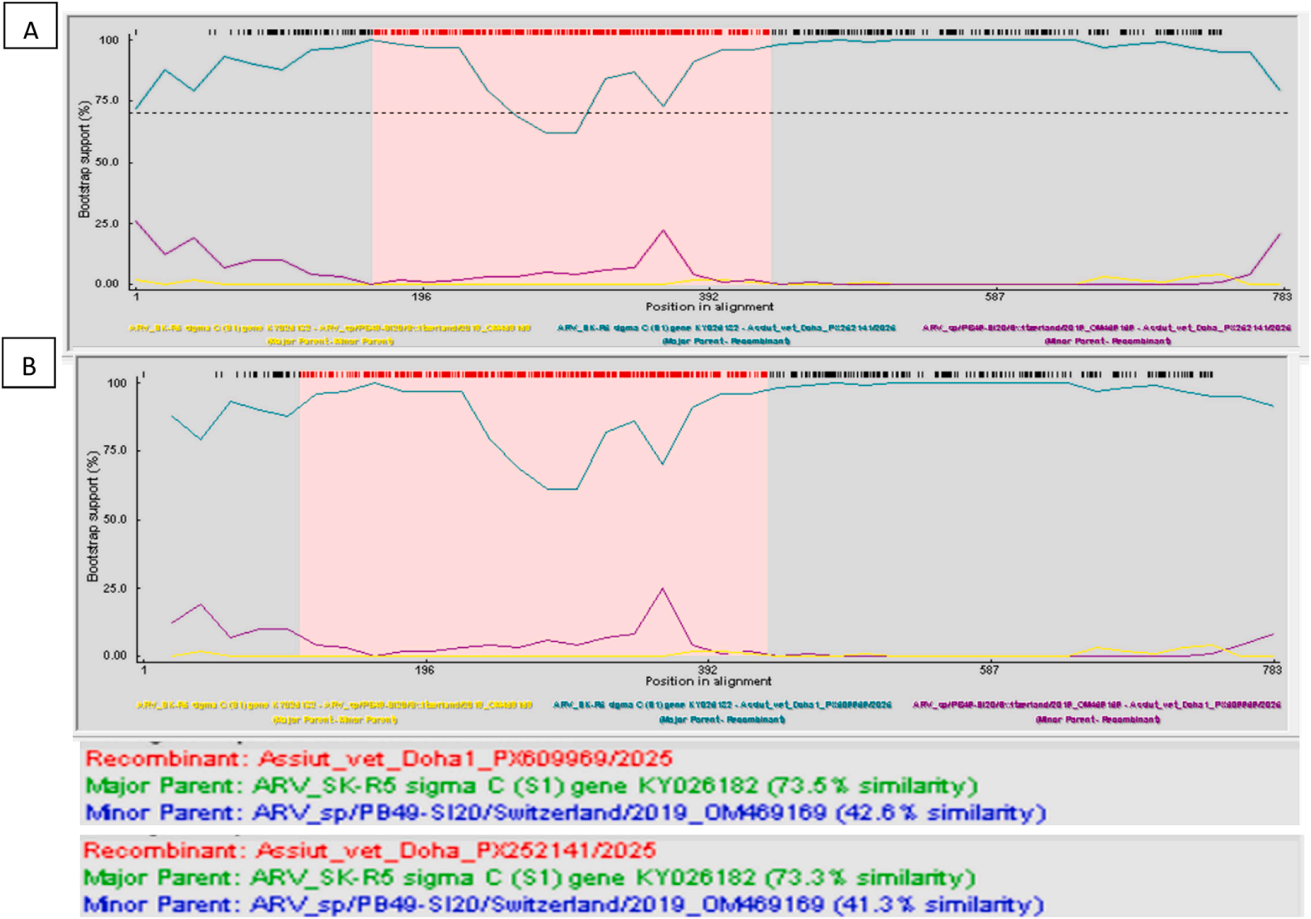
Recombination analysis of the Egyptian isolates: Assiut-vet-Doha (PX252141), and Assiut_vet_Doha1 (PX609969) performed using RDP4 software. (A, B): The recombinant region is indicated in pale pink. Potential parents involved in the recombination events as follows: the ARV_SK-R5 σC (S1) gene strain KY026182 of genotype V, from South Korea, is a major parent. ARV_sp/PB49-S120/Switzerland/2019 (OM489189) genotype II, from Switzerland, is a minor parent.

Phylogenetic analysis using the neighbor-joining method revealed that nine isolates (PX252141, PX609969–PX609974, PX609975, and PX609976) clustered within Genotype V (GCV), forming a distinct Upper Egypt subcluster closely related to Israeli and previous Egyptian field strains. Notably, two isolates (PX252143 and PX609972) grouped within Genotype IV (GCIV), representing the first report of this genotype in broiler flocks from Upper Egypt. All isolates were highly divergent from the S1133-like Genotype I vaccine strains, sharing only 49.2%–53.5% nucleotide and 38.4%–42.1% amino acid identity. These findings provide the first molecular evidence of the co-circulation of genotypes IV and V in Upper Egypt **(Figure 3).** Concerning recombination analysis using the RDP v.4.97 program using the BootScan and 3Seq, the Egyptian isolates Assiut-vet-Doha (PX252141/2025), and Assiut_vet_Doha1 (PX609869/2025) represented a potential recombinant strain of (P value = 5.67 × 10^–4^ and 8.87 × 10^–2^; respectively). The recombination events identified ARV_SK-R5 σC (S1) gene strain KY026182 of genotype V, from South Korea, as the major parental strain, showing 73.5% sequence similarity, while ARV_sp/PB49-S120/Switzerland/2019 (OM489189) genotype II, from Switzerland, was identified as the minor parental strain with 42.6% similarity. The estimated recombination breakpoint analysis was located at nucleotide positions 161 and 431 of the alignment (95% confidence interval) and positions 109 and 432 (95% confidence interval). The recombination signals were strongly supported across the highlighted genomic region, indicating that genetic exchange had occurred between the two parental lineages within the σC gene segment (Fig. 4, Fig. 3).

### 3.5 Nucleotide and amino acid identity and variation

Pairwise nucleotide identity among the 11 Upper Egyptian isolates ranged from 91% to 100%, indicating a common ancestral origin; however, these values dropped dramatically to 51%–53% when compared to classical vaccine strains such as S1133, 1733, and 2408. Multiple sequence alignment and phylogenetic tree construction revealed that the current isolates were distributed into two distinct clusters: Genotype V, which shared high similarity (92%–96%) with global reference strains, including USA, China, Israeli, Brazil, and Canada. In contrary, our genotype IV isolates showed low homology with their respective global reference variants (59-61% and 47-50%, respectively) based on nucleotide and amino acid identities. Both genotypes demonstrated significant divergence from Genotypes I, II, and III, with similarity values ranging only between 48% and 56%. Most importantly, the amino acid identity between these Upper Egyptian field isolates and the Genotype I vaccine strains was exceptionally low, ranging from 38% to 41% for Genotype V and 42% to 45% for Genotype IV, levels that are far below the threshold typically associated with robust cross-protection (Table 3 and Table S1, supplementary materials).

**Table 3:** Comparison of Nucleotide (NT) and Amino Acid (AA) sequence identities of Upper Egypt ARV isolates compared to reference and vaccine strains.

Comparison Groups	Nucleotide (NT) identity Range (%)	Amino Acid (AA) identity Range (%)
**Within Upper Egypt isolates (n=11)**	85-99%	85–100%
**Upper Egypt isolates vs. Genotype I (Vaccines: S1133, 1733, 2408)**	52-53.5%	38.4–42.1%
**Upper Egypt isolates vs. Genotype IV reference strains**	59-61%	47–50%
**Upper Egypt isolates vs. Genotype V reference strains**	92-94%	91–96%
**Genotype IV isolates (PX252143, PX609972) vs. Genotype V isolates**	86-90%	85–90%

### 3.6 Nucleotide and amino acid substitutions

Nucleotide and amino acid substitutions were determined by comparing the σC gene sequences of the 11 Upper Egyptian field isolates with the classical S1133 reference strain (Genotype I; GCI)**,** which is widely used as the parental strain of commercial *Avian orthoreovirus* vaccines. Alignment of the σC gene revealed 48 nucleotide substitutions relative to the S1133 strain, with 17 (35.4%) being nonsynonymous. Most substitutions were concentrated in the hypervariable receptor-binding region [amino acid (aa) 180–270]. Key amino acid replacements included Q201R, T220I, N235D, and Y247H, which are located within domains critical for cell attachment and viral neutralization. These sequence differences further highlight the marked divergence between the circulating field isolates and the classical Genotype I vaccine strain **(Figures S1, Figure S2, supplementary material).**

### 3.7 Pathogenicity and performance impact of the isolated ARV strain

**Fig. 5:**
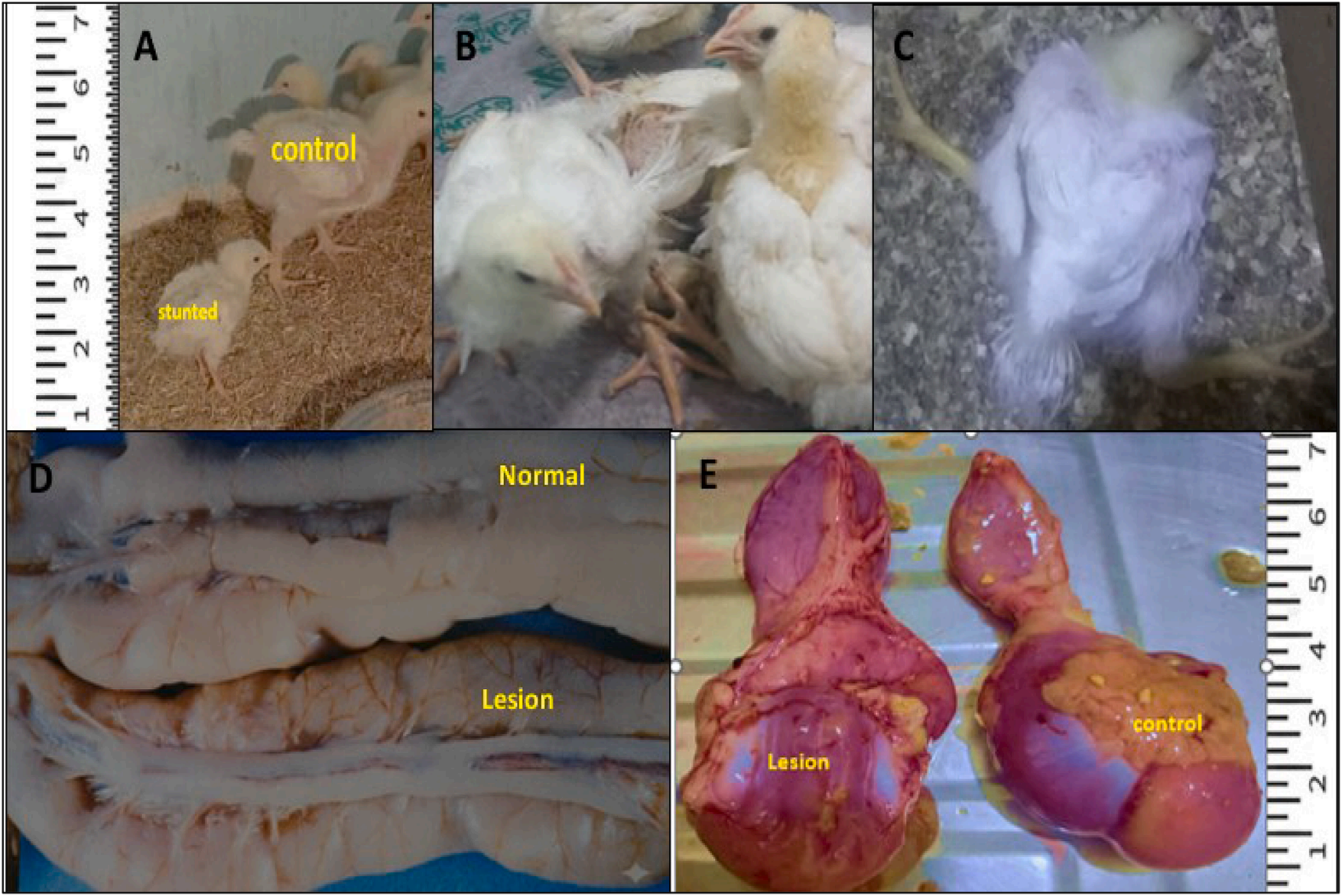
Clinical and gross lesions in broiler chicks experimentally infected with the field Avian orthoreovirus isolate. (A) Stunted chicks showing marked growth retardation compared with controls;(B) Abnormal “helicopter-like” feathering; (C) Chick unable to stand due to severe arthritis and tendon inflammation. (D) Marked atrophy of the pancreas and (E) Enlarged proventriculus compared with normal organs.

Clinically, inoculated SPF chicks showed clinical signs starting at 5 days post-infection (dpi), including reduced activity and lameness. By 14 dpi, stunting and “helicopter feathering” were prominent. Necropsy at 42 dpi revealed serous synovitis, tendon thickening, pancreatic atrophy, and proventriculitis**.** The SPF chickens exhibited swelling and edema in the footpads after the viral inoculation with a morbidity rate of 100% (Fig. 5 A-E). In contrast, no clinical signs or mortality were noticed in the control group.

**Fig. 6:**
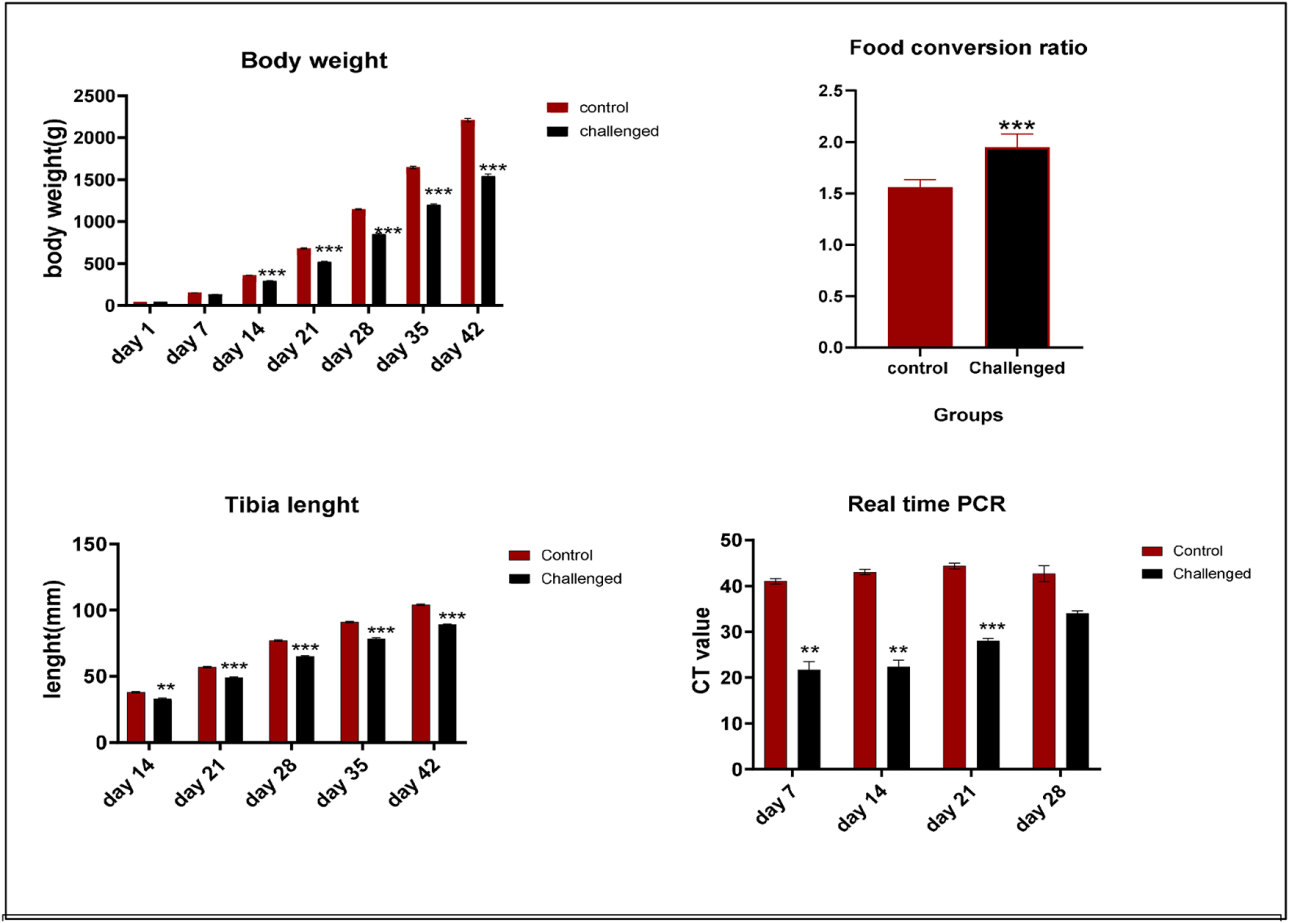
Comparison of body weight, feed conversion ratio (FCR), tibial bone length, and viral load in tendon (RT-qPCR) between control and challenged broiler groups. Values are expressed as Mean ± SE (n = 3). Statistical significance is indicated by asterisks: * (p < 0.05) represents significant, ** (p < 0.01) represents highly significant, and *** (p < 0.001) represents very highly significant differences between groups.

The distribution of clinical manifestations is summarized in Table 4. Arthritis/tenosynovitis was observed in 60% (18/30) of the challenged birds, while growth retardation was recorded in 80% (24/30) of the group. No clinical signs were observed in the control group (0). The impact on growth performance was profound (Table 5**).** Infected chicks showed a significant reduction in body weight starting from 7 dpi till 21 dpi (p < 0.01), which was significantly lower than that of the control group. Also, there is a marked increase in the feed conversion ratio (FCR) by the end of the study (p <0.05) compared to controls (Fig. 6). Additionally, skeletal development was impaired, as evidenced by significantly shorter tibia lengths in the infected group (p < 0.01).

**Table 4:** Temporal progression of clinical signs and mortality in broiler chickens experimentally challenged with the Egyptian ARV strain.

Clinical Signs/Days	Group	Day 7	Day 14	Day 21	Day 28	Day 35	Day 42
Arthritis/Tenosynovitis	Control	0/30	0/25	0/20	0/15	0/10	0/5
	Challenged	30/2	24/5	18/8	13/10	9/9	5/5
Growth Retardation	Control	0/30	0/25	0/20	0/15	0/10	0/5
	Challenged	0/30	4/24	9/18	11/13	9/9	5/5
Mortality	Control	0/30	0/25	0/20	0/15	0/10	0/5
	Challenged	1/30	0/24	1/19	0/14	0/10	0/5

**Table 5:** Effect of viral challenge on body weight and feed conversion ratio in broilers at different time points.

	Body weight (g)			feed conversion ratio (FCR)		
*Days*	control	challenged	P value	Control	Challenged	P value
1^b^	42±0.3333	41.7±0.3333	<0.983	—	—	
7^a^	150±0.300	130±0.300	<0.0011	—		
14^a^	359±3.930	291.7±3.930	<0.0003	—	—	
21^a^	680±0.500	520±0.500	<0.0002	—	—	
28^a^	1147±3.333	850±3.333	<0.0001	—		
35^a^	1647±3.333	1200±3.333	<0.0005	—	—	<0.0306
42^a^	2210±8.819	1543±8.819	<0.0001	1.558±0.075	1.95±0.1291	

Different superscript letters (a, b) indicate significant differences between groups at the different time point (P < 0.05). Data are presented as mean ± SE.

Quantitative RT-PCR confirmed active and sustained viral replication. Significantly lower Ct values (indicating higher viral loads) were detected in the tendons of infected birds (p < 0.01) (Table 6; Fig.s 6). To ensure the specificity of these findings, all specimens were confirmed negative for the differential viral and bacterial pathogens (Table 1).

**Table 6:** Effect of viral challenge on viral load (RT-PCR) and tibial bone growth in broilers at different time points.

	Real time PCR in tendon			Tibia length(mm)		
Days	Control	Challenged	P value	Control	Challenged	P value
7^a^	41.00±1.280	34±1.280	<0.0021	—	—	—
14^a^	43±1.280	28±1.280	<0.0031	38±0.8692	33±0.8692	<0.003
21^a^	44±1.280	22.33±1.280	<0.0002	57±0.8692	49±0.8692	<0.001
28	36±1.280	21.67±1.280	0.4946	77±0.8692	65±0.8692	<0.0004
35^a^	**—**	—	—	91±0.8692	78±0.8692	<0.0005
42^a^	**—**	—	—	104±0.8692	89±0.8692	<0.0001

Different superscript letters (a, b) indicate significant differences between groups at different time point (P < 0.05). Data are presented as mean ± SE.

**Note:** Numbers represent (affected birds / total remaining birds). The cumulative incidence of arthritis and growth retardation throughout the experiment was 60% (18/30) and 80% (24/30), respectively.

## 4 Discussion

Over the past decade, poultry health has been increasingly challenged by different viral diseases in Egypt; causing numerous epidemics (Shosha et al., 2024**;** Shosha et al., 2025c). *Avian orthoreovirus* remains a major threat to the Egyptian poultry industry, causing severe economic losses despite intensive vaccination programs. Over the past decade, a significant "knowledge gap" has emerged regarding the evolving genetic landscape of ARV in Upper Egypt. This study was conducted to fill this gap, providing the first comprehensive molecular and pathological characterization of the currently circulating variants in this region to guide more effective control strategies. The clinical presentation in Upper Egyptian flocks, characterized by lameness, 'helicopter-like' feathers (a pathognomonic marker of nutrient malabsorption), and stunted growth, aligns perfectly with the established descriptions of viral arthritis and runting-stunting syndrome (RSS) (Jones, 2000; Sallam et al., 2025). The appearance of these abnormal feathers is intrinsically linked to the ARV-induced pancreatic atrophy and intestinal damage observed in our study, which leads to the malabsorption of essential nutrients required for normal feather development. This clinical consistency proves that despite the genetic evolution of ARV, its tropism for synovial membranes and the digestive tract remains a stable pathogenic feature. Furthermore, while genotypes IV and V have been recently reported in Northern Egypt, our findings provide the first evidence of their established co-circulation in Upper Egypt governorates, suggesting a significant nationwide geographical expansion of these variants. Interestingly, our isolation rate of 30% is notably higher than the 18% reported in Northern Egypt (Lu et al., 2015), a discrepancy that likely stems from the high density of backyard rearing and potential biosecurity gaps in Upper Egypt, which facilitate environmental persistence and rapid transmission of these emerging genotypes.

The successful recovery of these isolates via the yolk sac of SPF eggs, followed by characteristic embryo lesions, confirms the viability and high infectivity of these strains, echoing the gold-standard protocols that suggest the yolk sac route is highly sensitive for recovering variants that might be missed by other methods (Gamble and Sellers, 2022).

The phylogenetic analysis of the σC gene revealed a significant epidemiological shift, as our isolates clustered into Genotypes IV and V. While recent surveillance in Northern Egypt and the Nile Delta has begun to identify these genotypes (Mohamed et al., 2020), to our knowledge, this study provides the first documentation of the co-circulation of Genotypes IV and V in Upper Egypt governorates during 2024–2025. This finding is consistent with the global expansion of non-S1133 genotypes observed in the Middle East and Asia (Gallardo, 2022), but it diverges from older Egyptian studies that primarily identified Genotype I. This emergence reflects a geographical expansion of these variants across Egypt and suggests a 'genotypic replacement,' where the continuous use of classical Genotype I vaccines has created an immunological environment that selects for Genotype IV and V variants, which appear more adapted to survive under such vaccination pressure.

Furthermore, the phylogenetic proximity between the Egyptian Genotype V isolates identified in this study and Israeli strains indicates a shared regional evolutionary lineage within the Middle East. This genetic clustering suggests potential transboundary movement or environmental diffusion of the virus, possibly facilitated by migratory bird flyways or the interconnected poultry trade routes between regions. The presence of these closely related sub-lineages in Upper Egypt reinforces the hypothesis that these variants are successfully diffusing southward, highlighting the necessity for regionally coordinated surveillance and the development of vaccines that address the specific antigenic landscape of the Middle East.

Regarding genetic similarity, while our isolates showed high internal homogeneity (91%–100%), they shared a remarkably low nucleotide identity (52%) with classical vaccine strains (S1133, 1733, and 2408). More critically, the amino acid identity ranged from 38%–45%, indicating divergence within the immunologically important σC protein as it is the main neutralizing antigen and the most variable protein in ARV (Franzo et al., 2024). Since an amino acid identity of at least 80% is typically required for robust cross-protection (Liu et al., 2023). Our results provide a clear scientific explanation for the recurring outbreaks; the circulating field variants are so genetically distant from Genotype I vaccines that they are likely not "recognized" by the vaccine-induced antibodies, rendering current control programs largely ineffective. Therefore, the marked sequence divergence observed suggests a potential antigenic mismatch between the circulating field isolates and Genotype I vaccine strains. However, sequence homology alone cannot establish antigenic escape or reduced vaccine efficacy, and these molecular findings should be interpreted cautiously until confirmed by cross-virus neutralization assays and heterologous vaccination-challenge studies using representative local strains. The recombination analysis identified putative genetic exchange within the σC gene of two Egyptian ARV isolates, with the closest related parental sequences belonging to genotype V from South Korea and genotype II from Switzerland. Although these findings do not indicate direct transmission from either country, they highlight the genetic relatedness of the Egyptian isolates to ARV lineages circulating in different geographic regions. This observation supports the concept that the global movement of poultry, breeding stocks, hatching eggs, or other poultry-associated materials may facilitate the dissemination and co-circulation of genetically distinct ARV lineages. Several studies indicate that ARV lineages are not confined to local poultry populations; instead, they circulate through long-distance viral migrations and co-circulation of genetically diverse strains across countries (Farkas et al., 2016; Zhang et al., 2021; Nour et al., 2023). Moreover, when different viruses co-infect the same host population, recombination can generate novel genetic variants that may influence viral evolution, host adaptation, and antigenic diversity. Therefore, the detection of putative inter-genotype recombination in the present study emphasizes the importance of continuous molecular surveillance and international genomic monitoring to track the emergence and spread of recombinant ARV strains and to improve the effectiveness of disease control strategies.

The identification of key substitutions in the σC hypervariable region (aa 180–270), such as Q201R and Y247H, agrees with structural models identifying these positions as vital for receptor binding and neutralizing antibody interaction (Goldenberg et al., 2010). Previous studies have shown that mutations within the hypervariable receptor-binding domain have been associated with altered receptor-binding properties, modify exposed antigenic epitopes, and reduced cross-neutralization between divergent ARV genotypes (Kant et al., 2003; Liu et al., 2003; Lu et al., 2015; Chen et al., 2025). Consequently, the accumulation of amino acid substitutions observed in the current Upper Egyptian isolates may contribute to antigenic drift and could reduce cross-protection against genotype I-derived vaccine strains. This molecular divergence is a direct consequence of long-term immune pressure, mirroring the evolution of variant ARVs in other poultry-producing regions, where viruses must continuously mutate to escape host immunity (Sellers, 2017). Nevertheless, the impact of these amino acid substitutions on antibody recognition and vaccine-induced immunity cannot be inferred solely from sequence analysis and requires confirmation through virus-neutralization assays and controlled vaccination-challenge experiments.

Finally, the experimental challenge confirmed the clinical relevance of our molecular findings, as the significant reduction in body weight gain, increased FCR, characteristic swelling of the footpads, and shortened tarsometatarsal bone length confirm the high virulence of these Genotype IV variants. These performance deficits align with established pathogenicity models (Rafique et al., 2024; Chen et al., 2025), where pancreatic atrophy and intestinal distension provide the biological basis for severe malabsorption. Interestingly, our isolates caused significant joint and enteric pathology without high mortality, which differs from acute outbreaks. This suggests that Egyptian strains have evolved a "subclinical persistence" strategy, maximizing economic damage through chronic growth retardation and lameness rather than bird loss, which allows the virus to circulate longer and spread more broadly within the poultry production system. Specifically, quantitative real time PCR demonstrated markedly elevated viral RNA levels in tendons, indicating that tendons represent the primary target organ of ARV replication. These findings are consistent with earlier reports by studies (Jiang et al., 2021; Yan et al., 2021).

Although this study provides critical insights into the Avian orthoreovirus landscape in Upper Egypt, certain limitations should be addressed in future research. The relatively small number of sequenced isolates warrants broader longitudinal surveillance and large-scale molecular epidemiology to fully map the viral evolution across various Egyptian governorates. Furthermore, integrating advanced molecular tools, such as whole-genome sequencing (WGS), would provide a deeper understanding of potential reassortment events between segment types.

While our study focused on gross pathology and performance, future investigations incorporating detailed histopathological and immunohistochemical (IHC) scoring of the pancreas, intestines, and tendons are essential to quantify the cellular impact of these variant genotypes. Furthermore, these results highlight the need to continuously evaluate whether currently circulating Egyptian ARV strains remain antigenically compatible with commercially available genotype I-based vaccines. Most importantly, these findings underscore the urgent necessity of developing future autogenous or regionally adapted vaccines that specifically incorporate the currently circulating Egyptian Genotype IV and V strains through comprehensive antigenic characterization. Given the documented co-circulation and high antigenic divergence, a bivalent autogenous vaccine approach may be the most viable strategy to overcome the limitations of commercial Genotype I-based vaccines. Continuous molecular monitoring, combined with *in vitro* cross-neutralization assays, remains indispensable for guiding effective vaccination programs and safeguarding the poultry industry against the evolving threat of Avian orthoreoviruses.

Finally, a limitation of the current study is that the *in vivo* pathogenicity and performance impact assessment was restricted to a single novel Genotype IV ARV strain. While this strain was selected as a representative isolate, further comparative studies including Genotype V strains are warranted to comprehensively elucidate and contrast the differential virulence and immunopathogenic profiles of these co-circulating genotypes in Egypt. Additionally, future surveillance should employ systematic random sampling to obtain representative estimates of flock-level ARV prevalence and strengthen epidemiological assessments and disease control strategies.

## 5 Conclusion

This study provides a comprehensive molecular and phylogenetic characterization of ARV Genotypes IV and V circulating in broiler chickens from Upper Egypt. Our findings reveal that these field strains exhibit significant genetic and antigenic divergence from existing Genotype I vaccine strains, which may explain the reported vaccination breakthroughs. The demonstrated pathogenic potential of these isolates in vaccinated progeny emphasizes that current control programs face challenges from emerging variant strains. Consequently, there is an urgent need to consider the development of regionally adapted or bivalent vaccines and to maintain continuous genomic surveillance. Such measures are essential to mitigate the economic impact of ARV-associated diseases and to guide effective biosecurity and vaccination strategies in the Egyptian poultry industry.

## Funding

This work received no external funding.

## Ethics statement

The study was approved by the Institutional Animal Care and Use Committee (IACUC) of the Faculty of Veterinary Medicine, Assiut University, Egypt (Approval No. 06/2025/0374) on 20 October 2025, and was conducted in accordance with the ethical guidelines for animal welfare.

Samples were collected by members of this group.

## CRediT authorship contribution statement

**Doha Abd Alrahman Ahmed:** Writing – review & editing, Writing – original draft, Resources, Project administration, Methodology, Investigation, Funding acquisition, Formal analysis, Data curation, Conceptualization. **Shimaa Mohamed Ali Ahmed Khair:** Conceptualization. **Mohammed A. GamalEldin:** Data curation. **Ibrahim Mohamed Eldaghayes:** Methodology, Investigation, Funding acquisition, Formal analysis, Data curation, Conceptualization. **Eman Abd Elmenum Shosha:** Project administration, Methodology, Investigation, Funding acquisition, Formal analysis, Data curation, Conceptualization.

## Declaration of competing interest

The authors declare that they have no known competing financial interests or personal relationships that could have appeared to influence the work reported in this paper.

## Data Availability

All data generated or analyzed in this study are available from the corresponding author on reasonable request.

## Appendix Supplementary materials

Image, application 1
