# Supplementary material for: Pathogenicity and molecular evolutionary analysis of novel Avian orthreovirus genotypes IV and V in Upper Egypt
Source: Virus Res. 2026 Jul 20;371:199779. doi: 10.1016/j.virusres.2026.199779 (PMC13452221; doi:10.1016/j.virusres.2026.199779)
Supplement: Supplementary file 1 [file mmc1.docx]

**Table 4 S1 supplementary.** Pairwise nucleotide and amino acid identity (%) among Egyptian *Avian reovirus* isolates and representative global reference strains based on partial σC gene sequences.

**Figure S1(A):** Deduced amino acid sequence alignment of the σC protein among Egyptian isolates and reference ARV strains.

**Figure S1 (B):** Deduced amino acid sequence alignment of the σC protein among Egyptian isolates and reference ARV strains.

**Figure S2 (A).** Multiple Alignment of nucleotide sequences of the eleven Egyptian isolates with representative reference strains from different genotypes (I–VI). Panel (A) represents the alignment from nucleotide position 1 to 240. Dots (.) indicate conserved nucleotides, while colored letters represent substitutions

**Figure S2** (B)**.** Multiple Alignment of nucleotide sequences of the eleven Egyptian isolates with representative reference strains from different genotypes (I–VI). Panel (B) represents the alignment from nucleotide position 250 to 480. Dots (.) indicate conserved nucleotides, while colored letters represent substitutions

**Figure S2** (C)**.** Multiple Alignment of nucleotide sequences of the eleven Egyptian isolates with representative reference strains from different genotypes (I–VI). Panel (C) represents the alignment from nucleotide position 490 to 720. Dots (.) indicate conserved nucleotides, while colored letters represent substitutions

**Figure S2** (D)**.** Multiple Alignment of nucleotide sequences of the eleven Egyptian isolates with representative reference strains from different genotypes (I–VI). Panel (D) represents the alignment from nucleotide position 730 to 784. Dots (.) indicate conserved nucleotides, while colored letters represent substitutions
